# Effect of *Helicobacter pylori* eradication evaluated using magnifying endoscopy with narrow-band imaging in mixed-type early gastric Cancer

**DOI:** 10.1186/s12876-023-03064-z

**Published:** 2023-12-04

**Authors:** Yoshitaka Tokai, Yusuke Horiuchi, Noriko Yamamoto, Ken Namikawa, Shoichi Yoshimizu, Akiyoshi Ishiyama, Toshiyuki Yoshio, Toshiaki Hirasawa, Junko Fujisaki

**Affiliations:** 1 Department of Gastroenterology, The Cancer Institute Hospital of Japanese Foundation for Cancer Research, Tokyo, Japan; 2 Department of Pathology, The Cancer Institute Hospital of Japanese Foundation for Cancer Research, Tokyo, Japan

**Keywords:** Endoscopy, *Helicobacter pylori*, Stomach neoplasm, Eradication therapy, Gastric cancer

## Abstract

**Background:**

The effect of *Helicobacter pylori (H.pylori)* eradication therapy on mixed-histological-type gastric cancer remains unclear. This study aimed to clarify the effect of *H. pylori* eradication therapy on mixed-histological-type early gastric cancer using endoscopic and histological findings.

**Methods:**

This single-center, retrospective study included patients with mixed-histological-type gastric cancer who underwent endoscopic submucosal dissection at the Cancer Institute Hospital. We compared detailed magnifying endoscopy with narrow-band imaging findings between eradicated and non-eradicated groups of patients with differentiated-type- and undifferentiated-type-predominant cancers. Subsequently, we performed histological evaluations of the non-cancerous epithelium covering differentiated-type components.

**Results:**

A total of 124 patients with mixed-type early gastric cancer were enrolled (eradicated group: 62 differentiated-type-predominant cancer patients and 8 undifferentiated-type-predominant cancer patients; non-eradication group: 40 differentiated-type-predominant cancer patients and 14 undifferentiated-type-predominant cancer patients). Regarding differentiated-type-predominant cancer, differentiated-type findings were detected in all patients in eradicated and non-eradicated groups. The difference in the detection rate of undifferentiated-type findings between both groups was not significant in differentiated-type-predominant cancer patients. In differentiated-type-predominant cancers, the percentage of non-cancerous epithelium covering differentiated-type components was higher in the eradicated group than in the non-eradicated group (median: 60% vs. 40%, *p* < 0.001).

**Conclusions:**

Although the pathological findings of differentiated-type-predominant cancer were affected by *H. pylori* eradication, eradication did not affect the diagnosis of differentiated-type-predominant early gastric cancer using magnifying endoscopy with narrow-band imaging. ME-NBI is useful for the early detection of D-MIX EGCs and diagnosis of histological types during endoscopy, regardless of whether *H. pylori* eradication therapy has been administered.

**Supplementary Information:**

The online version contains supplementary material available at 10.1186/s12876-023-03064-z.

## Background

*Helicobacter pylori* (*H. pylori*) infections are strongly correlated with gastric cancer development [1], and several reports have shown that *H. pylori* eradication reduces the incidence of gastric cancer [2]. Consequently, *H. pylori* eradication therapy is widely performed in Japan, leading to an increase in the number of post-eradication cases. However, early gastric cancers (EGCs) are often detected even after successful *H. pylori* eradication [3].

According to the Japanese gastric cancer treatment guidelines [4], tumor biopsy specimens and endoscopically resected tumors are histologically classified into either differentiated-type (D-type) or undifferentiated-type (U-type) tumors. In mixed-type EGCs, tumors consisting of both D-type and U-type components are classified into two types according to the quantitative predominance of one differentiation type.

In recent years, magnifying endoscopy with narrow-band imaging (ME-NBI) has been developed, and its diagnostic performance is reported to be superior to that of conventional endoscopy [5–7]. Therefore, in the guidelines of the Japan Gastroenterological Endoscopy Society, ME-NBI has become the standard examination for gastric cancer diagnosis [8].

In pure D-type EGCs, lesions are reportedly flattened after *H. pylori* eradication; as such, it is reported that the lateral extents of these EGCs are difficult to detect and diagnose despite using ME-NBI [9–11]. This occurs because a normal columnar epithelium sometimes appears over tumor tissue following successful eradication therapy [9]; this phenomenon makes lesion borderlines indistinct or results in a lack of obvious cancerous characteristics. In contrast, a previous study reported that *H. pylori* eradication improves pathological inflammatory cell infiltration and results in an easy diagnosis of demarcation in patients with pure U-type EGC using ME-NBI [12]. However, the effects of *H. pylori* eradication on endoscopic mixed-type EGC and histological findings have not been reported to date.

Some reports indicate that mixed-type EGC has a higher malignancy potential than other EGCs, including a high risk of lymph node metastasis [13] and a low rate of curative resection [14, 15]. Therefore, mixed-type EGCs should be detected at an early stage using an accurate endoscopic diagnostic procedure.

Hence, we aimed to clarify the effect of *H. pylori* eradication therapy on mixed-histological-type EGC using histological and ME-NBI endoscopic findings.

## Methods

### Patients

This single-center, retrospective study included patients initially treated using endoscopic submucosal dissection (ESD) between March 2005 and March 2018 at the Cancer Institute Hospital. The inclusion criteria were as follows: patients with complete en bloc resection to examine all specimens resected by ESD; and patients with mixed-type EGC and a history of *H. pylori* infection. The exclusion criteria were as follows: pure D-type or U-type EGC; unclear history of *H. pylori* eradication; EGC detected within 1 year after successful eradication [16]; recurrent lesions after ESD, poor image quality due to bleeding, halation, blur, or defocusing; and cases without high magnification images. Herein, we considered *H. pylori* cases to be non-eradicated when a urea breath test (Otsuka, Tokushima, Japan; ≥2.5‰ indicates positivity) and/or a serum anti-*H. pylori* antibody test (Eiken, Tokyo, Japan; ≥10 U/mL indicates positivity) yielded positive results [17], without a history of *H. pylori* eradication. The eradicated cases were defined as follows: for cases in which eradication therapy was performed in our hospital, successful eradication was confirmed in a urea breath test ≥1 month post-therapy [17]. In cases where eradication therapy was previously performed in another hospital, successful eradication was confirmed by asking the patient about successful eradication history, in addition to negative urea breath and/or serum anti-*H. pylori* antibody tests.

### Endoscopic procedures

Endoscopic images were taken within 1 month before endoscopic resection (ER) as a detailed examination or directly before ER. For the procedure, an endoscope tip with a soft black hood (MAJ-1990 for GIF-H260Z and MAJ-1989 for GIF-H290Z; Olympus, Tokyo) mount was used. EGCs were observed as follows. First, we observed the lesion with white light. Second, ME-NBI was performed. Finally, chromoendoscopy was performed using indigo carmine. Observations with ME-NBI were performed using the following steps. First, the demarcation between cancerous and non-cancerous areas was observed at lower magnification. Second, maximal magnification was used for qualitative diagnosis. Third, biopsies around the lesion were usually performed on all patients to confirm that the cancer was not spreading to surrounding areas. Finally, a biopsy from the cancerous area was performed if not previously performed at another hospital. Esophagogastroduodenoscopy (EGD) was performed by board-certified endoscopists of the Japan Gastroenterological Endoscopy Society or non-board-certified endoscopists under the supervision of endoscopists who were board-certified.

EGD images were captured using magnifying endoscopes (GIF-H290Z and GIF-H260Z; Olympus Medical Systems Co., Ltd., Tokyo, Japan) and standard endoscope video systems (EVIS LUCERA CV-260/CLV-260 and EVIS LUCERA ELITE CV-290/CLV-290SL; Olympus Medical Systems Co., Ltd., Tokyo, Japan).

### Definitions of histological findings

According to Japanese classifications of gastric carcinoma [18], the endoscopically resected tumors were histologically classified as D-type and U-type EGCs. D-type EGC included papillary adenocarcinoma (pap) and tubular adenocarcinoma (tub1: well-differentiated adenocarcinoma, tub2: moderately differentiated adenocarcinoma), while U-type EGC included poorly differentiated adenocarcinoma (por1, por2) and signet-ring cell carcinoma (sig). Pure D-type and pure U-type consisted of D-type EGC components and U-type EGC components, respectively. D-MIX EGC was defined as differentiated-type-predominant EGC containing undifferentiated-type components, and U-MIX EGC was defined as undifferentiated-type-predominant EGC containing differentiated-type components.

### Patient characteristics

The patients were classified according to age, sex, and lesion characteristics, which included location, median size, macroscopic type, ulcerative findings, main histological type, and inflammatory cell infiltration. The items between eradicated and non-eradicated groups were compared in patients with D-MIX and U-MIX EGCs.

Regarding inflammatory cell infiltration, we performed pathological analysis using resected ESD specimen. We randomly selected 4-5 points of normal surrounding mucosa in ESD specimen and evaluated histological neutrophil infiltration grades and mononuclear cell infiltration according to the updated Sydney classification [19]. The percentage of normal-to-mild infiltration was calculated and compared between non-eradicated and eradicated groups in patients with D-MIX and U-MIX EGCs.

### Evaluation using ME-NBI

**Fig. 1:**
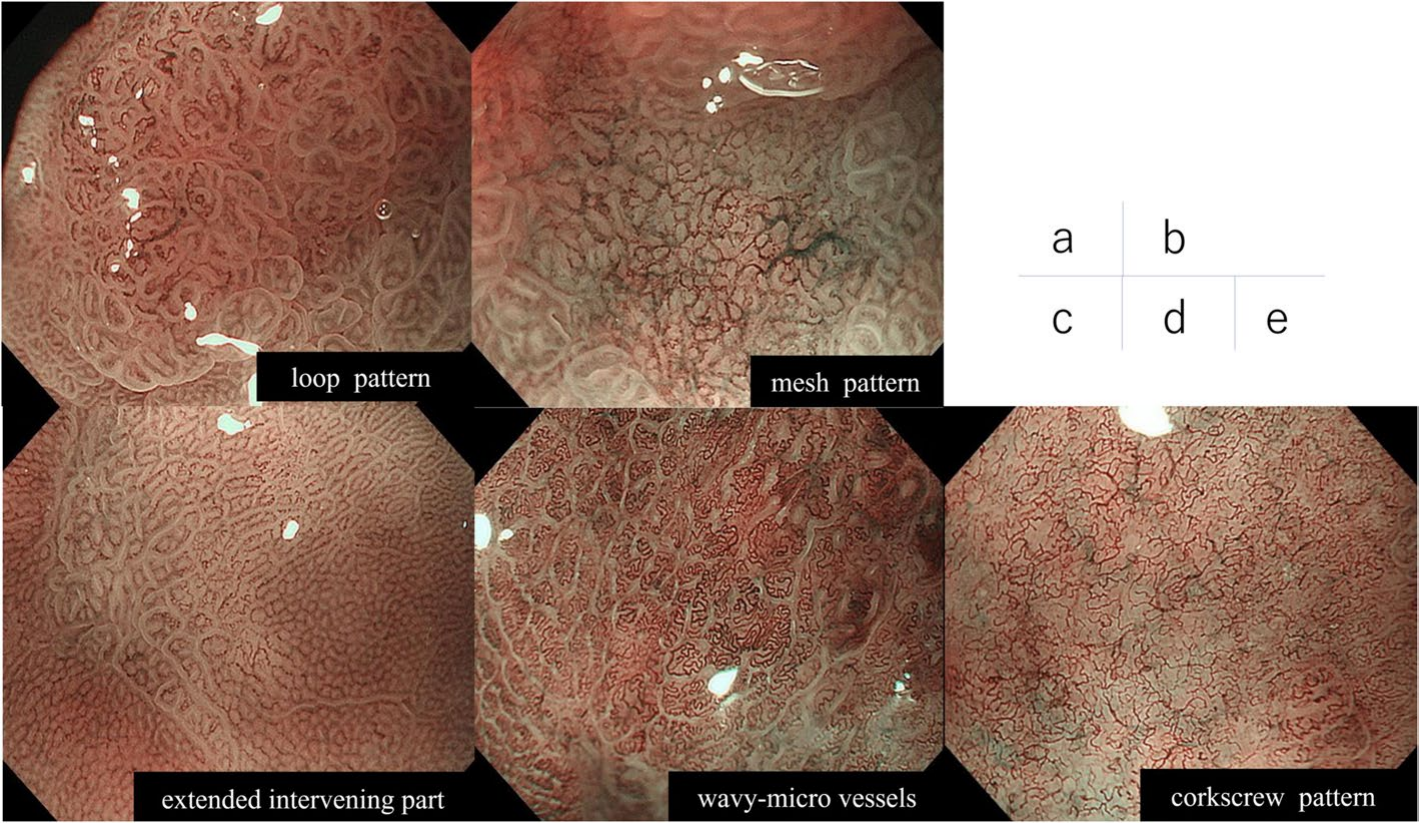
Magnifying endoscopy with narrow-band imaging findings of differentiated-type and undifferentiated-type gastric cancer. Regarding magnifying endoscopy with narrow-band imaging findings, we considered the loop and mesh patterns as findings of differentiated-type cancer (Fig. 1a, b), and the extended intervening part, wavy-microvessels, and corkscrew pattern as findings of undifferentiated-type cancer (Fig. 1c, d, e)

Two board-certified endoscopists of the Japan Gastroenterological Endoscopy Society (Y.T. and Y.H.) evaluated ME-NBI findings. Each endoscopist only knew the identification number of cases. Subsequently, these endoscopists extracted data from electronic medical records, and based on the previous images, judged whether there were D-type and U-type EGC in the ME-NBI findings. While evaluating the endoscopic findings, both endoscopists were blinded to the *H. pylori* eradication therapy history. For discrepancies in diagnoses, both endoscopists discussed the findings until a consensus was reached. ME-NBI findings of D-type and U-type EGCs were as follows. The endoscopists considered the loop and mesh patterns in ME-NBI findings as D-type EGC and the extended intervening part, wavy-microvessels, and corkscrew pattern in ME-NBI findings as U-type EGC [20–24] (Fig. 1). The mesh pattern was defined as connected microvessels with a mesh-like appearance [20], and the loop pattern was defined as loop-forming microvessels at the tips of tubule-like or villus-like mucosal structures, which were associated with branched pits [20, 22]. An extended intervening part was defined as widened spaces between crypts in the cancerous mucosa compared with the surrounding noncancerous mucosa [24, 25]. Wavy microvessels were defined as vessels with unconnected curves or spirals [23]. The corkscrew pattern was defined as isolated and disordered vessels [21].

We previously reported that while diagnosing the predominant histological type of mixed-type EGC, a mesh pattern was significantly more frequent in patients with D-MIX EGC than in those with U-MIX EGC [26]. Additionally, the corkscrew pattern was significantly more frequent in patients with U-MIX EGC than in those with D-MIX EGC [26]. To clarify the differences in these ME-NBI findings if *H. pylori* were eradicated, detailed ME-NBI findings between the eradicated and non-eradicated groups were compared in patients with D-MIX and U-MIX EGCs and specific D-type and U-type ME-NBI findings were clarified.

### Histological findings of the non-cancerous epithelium

In D-type EGC, the surface of the D-type component may be covered by epithelium with low-grade atypia [27] and non-neoplastic epithelium [11] after eradication. Conversely, as in “crawling-type” adenocarcinomas [28], the normal epithelium exists on the surface of the cancer, with or without eradication. Therefore, if the atypia of the superficial epithelium was weaker than the atypia of the deeper cancer, the epithelium was judged as non-cancerous epithelium (NCE).

**Fig. 2:**
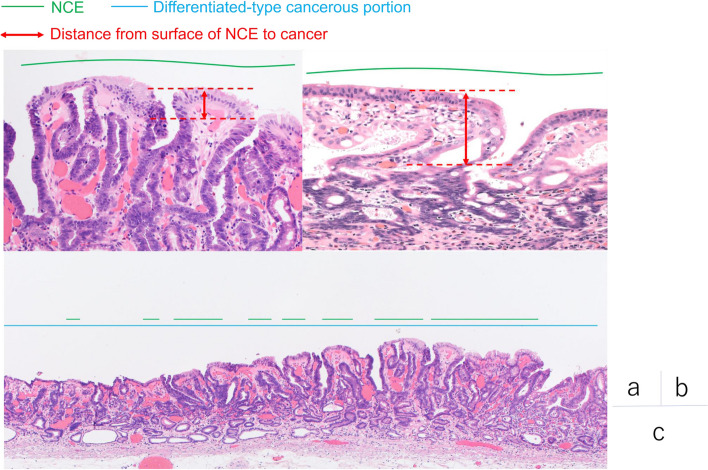
Definitions of non-cancerous epithelium (NCE) and “distance from the surface of the NCE to the cancer”. In case the atypia of the superficial epithelium was weaker compared with the atypia of the deeper cancer, we judged the epithelium as NCE (Fig. 2a, b green line). We measured the shortest distance from the surface of the NCE to the cancer, which existed just below the NCE in the mucosa, and defined it as the “distance from the surface of the NCE to the cancer” (Fig. 2a, b red arrow). We calculated the percentage of the differentiated-type cancerous portion covered by NCE in each patient. The NCE percentage was calculated as the length of the differentiated-type cancerous portion covered by NCE in all slides divided by the length of the differentiated-type cancerous portion in all slides (Fig. 2c)

NCE was evaluated using specimens stained with hematoxylin and eosin under the supervision of a pathologist specializing in the gastrointestinal tract (N.Y.). First, the shortest distance was measured from the surface of the NCE to the cancer that existed just below the NCE in the mucosa (hereafter called “distance from the surface of the NCE to the cancer”) (Fig. 2a, b). The measurement method was as follows: three slides per case were extracted, and the “distance from the surface of the NCE to the cancer” was measured in each slide. We calculated the average “distance from the surface of the NCE to the cancer” in each case from three slides. Next, the percentage of NCE was calculated as follows: the length of D-type components covered by NCE in all slides/the length of D-type components in all slides (Fig. 2c).

Finally, the percentage of NCE and “distance from the surface of the NCE to the cancer” were compared between D-MIX and U-MIX EGC patients with non-eradicated and eradicated *H. pylori* infections.

### Statistical analyses

A Fisher’s exact test was used to compare variables other than age and tumor diameter between eradicated and non-eradicated groups. Age, tumor size, percentage of NCE, and “distance from the surface of the NCE to the cancer” were calculated and analyzed using t-tests, and when data were not normally distributed, the Mann–Whitney U test was performed. A *p*-value < 0.05 was considered statistically significant. All statistical analyses were performed using EZR (Saitama Medical Center, Jichi Medical University, Saitama, Japan) [29].

## Results

**Fig. 3:**
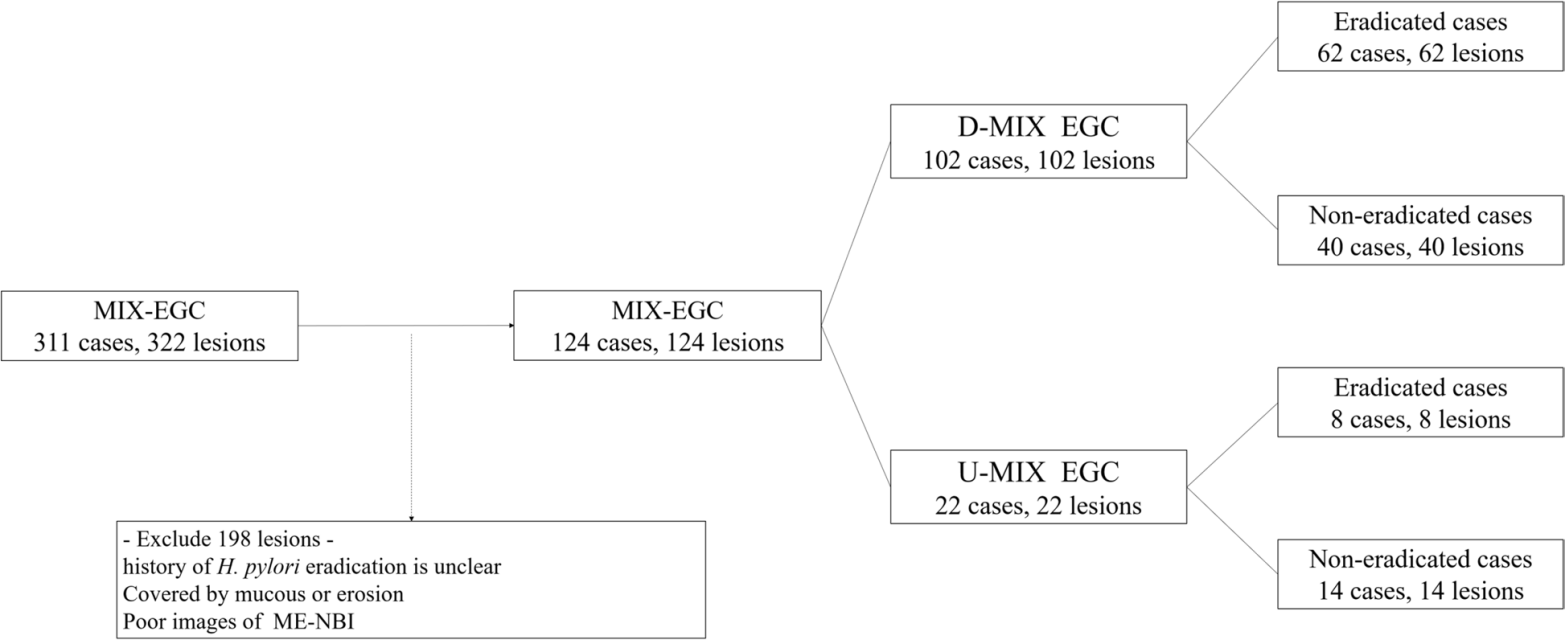
Patient flow diagram. Mixed-EGC, mixed-type early gastric cancer; D-MIX, differentiated-type predominant mixed type; U-MIX, undifferentiated-type predominant mixed type

A total of 322 MIX EGC lesions from 311 patients underwent ESD (Additional File 1). Among them, 198 lesions were excluded. Finally, a total of 124 patients with MIX-EGC were enrolled. Patients with MIX-EGC were divided into eradicated (62 D-MIX EGC patients, 8 U-MIX EGC patients) and non-eradiated groups (40 D-MIX EGC patients, 14 U-MIX EGC patients) (Fig. 3).

Regarding the characteristics of patients with D-MIX EGC (Table 1), the percentage of patients with tub2 was higher than that of tub1 in both non-eradicated and eradicated groups (70% vs. 77.4%, respectively). Regarding inflammatory cell infiltration, the percentage of normal-to-mild mononuclear cell infiltration and neutrophil infiltration was significantly higher in the eradicated group than in the non-eradicated group (82.3% vs. 22.5%, *p* < 0.0001 and 96.8% vs. 40%, *p* < 0.0001; respectively). Regarding the characteristics of patients with U-MIX EGC, the degree of inflammatory cell infiltration and percentage of normal-to-mild mononuclear cell infiltration and neutrophil infiltration were significantly higher in the eradicated group than in the non-eradicated group (100% vs. 21.4%, *p* = 0.001 and 100% vs. 21.4%, *p* = 0.001; respectively) (Table 2).

**Table 1:** Comparison of the characteristics between non-eradicated and eradicated groups in the differentiated-type predominant mixed-type early gastric cancers group

	Non-eradicated group *n* = 40	Eradicated group *n* = 62	*p*-value
**Age (years), median (range)**	65.5 (51–83)	69.5 (36–86)	0.26*
**Sex Male (%)**	29 (72.5%)	45 (72.6%)	> 0.99
**Location**			
**Upper**	7 (17.5%)	11 (17.7%)	0.48
**Middle**	15 (37.5%)	27 (43.6%)	
**Lower**	18 (45%)	21 (33.9%)	
**Remnant**	0 (0%)	3 (4.8%)	
**Median Size (mm), median (range)**	23.5 (7–100)	22 (4–70)	0.56*
**Macroscopic Type (%)**			
**0-IIa**	1 (2.5%)	2 (3.3%)	0.47
**0-IIb**	0 (0%)	1 (1.6%)	
**0-IIc**	34 (85%)	56 (90.3%)	
**Complex type**	5 (12.5%)	3 (4.8%)	
**Presence of ulcerative findings**	5 (12.5%)	14 (22.6%)	
**Invasion depth**			
**Mucosa**	29 (72.5%)	45 (72.6%)	> 0.99
**Submucosa**	11 (27.5%)	17 (27.4%)	
**Main histological type**			
**tub1**^a^	12 (30%)	14 (22.6%)	0.49
**tub2**^b^	28 (70%)	48 (77.4%)	
**Inflammatory cell infiltration (normal-mild)**			
**Neutrophils**	16 (40%)	60 (96.8%)	< 0.0001
**mononuclear cells**	9 (22.5%)	51 (82.3%)	< 0.0001

Data are presented as numbers (%)

^a^well-differentiated carcinoma; ^b^moderately differentiated adenocarcinoma

* Mann-Whitney U Test

**Table 2:** Comparison of the characteristics between non-eradicated and eradicated groups in the undifferentiated-type predominant mixed-type early gastric cancers

	Non-eradicated group *n* = 14	Eradicated group *n* = 8	*p*-value
**Age (years), median (range)**	73.5(40–82)	66 (56–72)	0.73*
**Sex Male (%)**	8 (57.1%)	4 (50.0%)	> 0.99
**Location**			
**Upper**	0 (0%)	1 (12.5%)	
**Middle**	10 (71.4%)	3 (37.5%)	0.23
**Lower**	4 (28.6%)	4 (50%)	
**Size (mm), median (range)**	21.5 (15–40)	21 (7–36)	0.59*
**Macroscopic Type (%)**			
**0-IIa**	0 (0%)	1 (12.5%)	
**0-IIc**	13 (92.9%)	6 (75%)	0.45
** complex type**	1 (7.1%)	1 (12.5%)	
**Presence of ulcerative findings**	4 (28.6%)	2 (25%)	> 0.99
**Invasion depth**			
**Mucosa**	11 (78.6%)	6 (75%)	> 0.99
**Submucosa**	3 (21.4%)	2 (25%)	
**Main histological type**			
**sig**^a^	2 (14.3%)	2 (25%)	0.60
**por**^b^	12 (85.7%)	6 (75%)	
**Inflammatory cell infiltration (normal-mild)**			
**Neutrophils**	3 (21.4%)	8 (100%)	0.001
**mononuclear cells**	3 (21.4%)	8 (100%)	0.001

Data are presented as numbers (%). ^a^signet ring cell carcinoma; ^b^poorly differentiated adenocarcinoma

* Mann-Whitney U Test

Subsequently, we compared the detection rate of cancerous findings by ME-NBI between non-eradicated and eradicated groups in the mixed-type EGC (Table 3). Regarding patients with D-MIX EGC, differentiated-type findings were detected in all patients in eradicated and non-eradicated groups, indicating no significant differences between both groups. In patients with U-MIX EGCs, the differences in the detection rate of undifferentiated findings between both groups were not significant.

**Table 3:** Comparison of detection rate of cancerous findings by magnifying endoscopy with narrow band imaging between non-eradicated and eradicated groups in the mixed-type early gastric cancers

	Cancerous findings	Non-eradicated group	Eradicated group	*p*-value
**D-MIX**^**a**^ **EGCs**^**c**^	Differentiated-type findings	40/40 (100%)	62/ 62 (100%)	> 0.99
	mesh pattern only	8 (20%)	17 (27.4%)	
	loop pattern only	7 (17.5%)	15 (24.2%)	
	mesh pattern + loop pattern	25 (62.5%)	30 (48.4%)	
	Undifferentiated-type findings	21/40 (52.5%)	33/62 (53.2%)	> 0.99
	extended	1 (2.5%)	4 (6.5%)	
	wavy	15 (37.5%)	19 (30.6%)	
	cork	2 (5%)	5 (8.0%)	
	2 of extended, wavy, cork	3 (7.5%)	4 (6.5%)	
	All (extended, wavy, cork)	0 (0%)	1 (1.6%)	
**U-MIX**^**b**^ **EGCs**	Differentiated-type findings	13/14 (92.9%)	6/8 (75%)	0.53
	mesh pattern only	3 (21.5%)	2 (25%)	
	loop pattern only	9 (64.3%)	3 (37.5%)	
	mesh pattern + loop pattern	1 (7.1%)	1 (12.5%)	
	Undifferentiated-type findings	11/14 (78.6%)	8/8 (100%)	0.27
	extended	2 (14.3%)	0 (0%)	
	wavy	2 (14.3%)	1 (12.5%)	
	cork	2 (14.3%)	2 (25%)	
	2 of extended, wavy, cork	5 (35.7%)	5 (62.5%%)	
	All (extended, wavy, cork)	0 (0%)	0 (0%)	

Data are presented as numbers (%). ^a^differentiated-type predominant mixed type; ^b^undifferentiated-type predominant mixed type; ^c^early gastric cancer, ^d^findings of extended intervening part; ^e^wavy-micro vessels; ^f^corkscrew pattern. EGCs: early gastric cancers

Furthermore, we clarified specific D-type and U-type ME-NBI findings in eradicated and non-eradicated groups by comparing ME-NBI findings between patients with D-MIX EGC and those with U-MIX EGC (Table 4). In the eradicated group, mesh patterns were observed significantly more often as D-type ME-NBI findings in patients with D-MIX EGC than in those with U-MIX EGC (76% vs. 38%, *p* = 0.038), and corkscrew patterns were observed significantly more often as U-type ME-NBI findings in patients with U-MIX EGC than in those with D-MIX EGC (75% vs. 18%, *p* = 0.0019).

**Table 4:** Comparison of magnifying endoscopy with narrow-band imaging findings between patients with differentiated-type predominant mixed type and patients with undifferentiated-type predominant mixed type

**Eradicated group**		D-MIX^a^ EGCs^c^ *n* = 62	U-MIX^b^ EGCs *n* = 8	*p*-value
**Differentiated-type findings**	mesh pattern	47 (76%)	3 (38%)	0.038
	loop pattern	45 (73%)	4 (50%)	0.23
**Undifferentiated-type findings**	extended	7 (11%)	1 (13%)	> 0.99
	wavy	26 (42%)	6 (75%)	0.13
	cork	11 (18%)	6 (75%)	0.0019
**Non-eradicated group**		D-MIX *n* = 40	U-MIX *n* = 14	*p*-value
**Differentiated-type findings**	mesh pattern	34 (85%)	4 (29%)	< 0.001
	loop pattern	32 (80%)	10 (71%)	0.49
**Undifferentiated-type findings**	extended	5 (13%)	2 (14%)	> 0.99
	wavy	13 (33%)	7 (50%)	0.34
	cork	6 (15%)	7 (50%)	0.025

^a^differentiated-type predominant mixed type; ^b^undifferentiated-type predominant mixed type; ^c^early gastric cancer

Even in the non-eradicated group, mesh patterns were significantly more observed as D-type ME-NBI findings in patients with D-MIX EGC than in those with U-MIX EGC (85% vs. 29% *p* < 0.001), and corkscrew patterns were observed significantly more often as U-type ME-NBI findings in patients with U-MIX EGC than in those with D-MIX EGC (50% vs. 15%, *p* = 0.025).

Then, we compared the proportion of NCE and “distance from the surface of the NCE to the cancer” between non-eradicated and eradicated groups in the mixed-type EGCs (Table 5). For D-MIX EGC, the percentage of NCE was higher in the eradicated group than in the non-eradicated group (median: 60% vs 40%, *p* = 0.00027). However, for U-MIX EGC, there were no significant differences between patients in non-eradicated and eradicated groups. Concerning the “distance from the surface of the NCE to the cancer,” there was no significant difference between the eradicated and the non-eradicated groups for both D-MIX EGC and U-MIX EGC.

**Table 5:** Comparison the percentage of the non-cancerous epithelium and “distance from surface of NCE to cancer” between non-eradicated and eradicated groups in the mixed-type early gastric cancers

	Histological findings	Non-eradicated group	Eradicated group	*p*-value
**D-MIX**^**a**^ **EGCs**^**c**^	Percentage of NCE, median (range)	40 (10–80)	60 (20–90)	0.00027*
	Distance from surface to cancer, μm, median (range)	55.5 (15–98)	62 (28–114)	0.30*
**U-MIX**^**b**^ **EGCs**	Percentage of NCE, median (range)	55 (20–70)	75 (40–100)	0.062*
	Distance from surface to cancer, μm, median (range)	53 (35–259)	64.5 (41–180)	0.61*

Data are presented as numbers (%). ^a^differentiated-type predominant mixed type; ^b^undifferentiated-type predominant mixed type; ^c^early gastric cancer

*Mann-Whitney U Test

## Discussion

This study clarified the effect of *H. pylori* eradication therapy on mixed-type EGC by comparing ME-NBI and histological findings between patients in eradicated and non-eradicated groups. To the best of our knowledge, the effect of eradication therapy on mixed-type EGC has not been reported yet.

In patients with D-MIX EGC, there was no significant difference in the detection rate of D-type ME-NBI findings between eradicated and non-eradicated groups. However, the percentage of NCE pathologically observed for the D-type component was significantly higher in the eradicated group than in the non-eradicated group.

The gastric capillary network that can be observed by ME-NBI is estimated to be approximately 100 μm from the mucosal surface layer [30]. This suggests that at depths of up to 100 μm, cancer may be detectable even if NCE is covering the surface of the cancer. Regarding the “distance from the surface of the NCE to the cancer,” in both D-MIX and U-MIX EGCs, it was approximately 60 μm for the eradicated and non-eradicated groups in this study. Thus, although the percentage of NCE after eradication was high, as previously reported for pure D-type EGCs [9, 10], the shallow “distance from the surface of the NCE to the cancer” did not affect the detection rate of D-type ME-NBI findings.

In patients with U-MIX EGC, there was no significant difference in the detection rate of U-type ME-NBI findings between the eradicated and non-eradicated groups. We previously reported that *H. pylori* eradication improves pathological inflammatory cell infiltration and results in easy diagnoses of demarcation in patients with pure U-type EGCs using ME-NBI [12]. This result is attributed to the improvements in neutrophil infiltration by eradication that improves the inflammation-induced extended intervening part of the normal mucosa in the background. Consequently, the contrast between the extended intervening part of cancer and the background mucosa becomes clear. However, in this study, the number of cases showing the extended intervening part was small, 13 and 14% in the eradicated and non-eradicated groups, respectively. Therefore, few cases were affected by *H. pylori* eradication. Hence, it is suggested that ME-NBI findings can be detected in for D-mix EGCs, regardless of the presence or absence of *H. pylori* infection. However, with regard to U-MIX, the small number of cases in this study may preclude such a conclusion.

In this study, mesh pattern ME-NBI findings were significantly more frequent in patients with D-MIX EGC, regardless of whether eradication was achieved. We previously reported that for diagnosing the main histological predominant component in EGC, the significant ME-NBI findings were a mesh pattern for D-MIX EGC and a corkscrew pattern for U-MIX EGC [26]. Therefore, it is possible to distinguish between D-MIX and U-MIX EGC, regardless of the eradication status. In patients with D-type EGC, ESD is indicated for intramucosal cancers without ulcers and intramucosal cancers within 3 cm with ulcerative findings. Conversely, in patients with U-type EGC, endoscopic treatment is indicated only for intramucosal cancers with a diameter ≤ 2 cm without ulcerative findings. Therefore, it is important to distinguish between D-MIX and U-MIX EGC, for which our results are valuable.

This study has several limitations. First, this was a single-center, retrospective study and there may have been an inherent selection bias. Second, the sample size of patients with U-MIX EGC may be insufficient. Finally, since we retrospectively collected ME-NBI images, we could not evaluate the effect of *H. pylori* eradication on mixed-type EGC demarcation diagnosis. Thus, further prospective multicenter studies assessing this matter by including a sufficient number of cases and comparing detailed ME-NBI and pathological findings are needed.

Despite these limitations, the results of this study provide supplementary data for detecting mixed-type EGCs. The findings are useful because they provide valuable information that may be the basis for future multicenter prospective studies.

## Conclusions

Although there was an effect on the pathological findings of D-MIX EGC, *H. pylori* eradication had no effect on D-MIX EGC diagnosis with ME-NBI. The use of ME-NBI is suggested to be useful for the early detection of D-MIX EGCs and diagnosis of histological types during endoscopy, regardless of whether eradication was performed.

### Supplementary Information

**Additional file 1.**

## Data Availability

The datasets generated during and/or analyzed during the current study are available from the corresponding author upon reasonable request.

## Abbreviations

D-type: differentiated-type
EGCs: early gastric cancers
EGD: esophagogastroduodenoscopy
ER: endoscopic resection
ESD: endoscopic submucosal dissection
ME-NBI: magnifying endoscopy with narrow-band imaging
NCE: non-cancerous epithelium
U-type: undifferentiated-type
