# Supplementary material for: Effect of Helicobacter pylori eradication evaluated using magnifying endoscopy with narrow-band imaging in mixed-type early gastric Cancer
Source: BMC Gastroenterol. 2023 Dec 4;23:425. doi: 10.1186/s12876-023-03064-z (PMC10694948; doi:10.1186/s12876-023-03064-z)
Supplement: Supplementary file 1 — Additional file 1. [file 12876_2023_3064_MOESM1_ESM.docx]

**Additional File 1.** Characteristics of 322 mixed-type early gastric cancers

|  | MIX-EGC  n=322 |
| --- | --- |
| Age (years), median (range) | 68 (36–91) |
| Sex, Male (%) | 239 (74.2%) |
| Location |  |
| Upper | 55 (17.1%) |
| Middle | 144 (44.7%) |
| Lower | 115 (35.7%) |
| Remnant | 8 (2.5%) |
| Size (mm), median (range) | 20 (4-100) |
| Macroscopic Type (%) |  |
| 0-IIa | 30 (9.3%) |
| 0-IIb | 1 (0.3%) |
| 0-IIc | 252 (78.3%) |
| complex type | 36 (11.1%) |
| 0-I | 3 (1%) |
| Presence of ulcerative findings | 43 (13.6%) |
| Invasion depth |  |
| Mucosa | 236 (73.3%) |
| Submucosa | 86 (26.7%) |
| Predominant type |  |
| Differentiated-type (D-MIX) | 251 (78.0%) |
| Undifferentiated-type (U-MIX) | 71 (22.0%) |

Data are presented as numbers (%)
